# Etiquette in the Context of Death and Dying: Communication and Conversation

**DOI:** 10.1177/00302228231196623

**Published:** 2023-11-03

**Authors:** Jennifer Dayes, Joseph Keenan, Michal Sadza, Karina Croucher

**Affiliations:** 1 Department of Psychology, 5289 Manchester Metropolitan University, Manchester, UK; 2 Archaeological and Forensic Sciences, 1905 University of Bradford, Bradford, UK

**Keywords:** etiquette, death, bereavement, thematic analysis, disenfranchised grief

## Abstract

Death, bereavement, and grief are experiences suffused with conflict and disenfranchisement. Intricately connected is ‘etiquette’ – the sense of ‘should’ ‘must’ ‘right’ ‘wrong’ ‘appropriate’ and ‘inappropriate’ individuals feel in death and bereavement situations. This paper is the first of two answering the question, ‘where does etiquette arise in death and bereavement situations and what does this ‘look like?’’ The theme *The etiquette of communication and conversation* is described, highlighting the importance of early communication for resolving conflict, what is considered ‘appropriate’ communication and support, and the social values underpinning these. Data highlighted how the CBT concept of ‘shoulding and musting’ manifests in death and bereavement situations, gave insight into etiquette’s role in disenfranchising grief through shaping conversations, and offered suggestions for bereavement support. Though the term ‘etiquette’ may be misleading out of context, the concept resonated with the bereaved community and provided language to discuss the nuances of their experiences.

## Introduction

### Death, Bereavement, and Grief in the West

Approximately 15,980,518 people died in developed countries in the year 2020 (Eurostat, 2021) and it is thought that in the United Kingdom, 72% of individuals had experienced bereavement in the last five years (Sue Ryder, 2019). According to the US Census Bureau (2021), there are 2.6 million deaths per year in the USA, creating an average of five grievers per death, and in a study based on Australian and Irish bereavement, an estimated 87% of individuals had been bereaved (Aoun, et al., 2020). Although there are issues with defining specific cultures, i.e., the problem of drawing a line between one culture and another (McLean et al., 2017), it is important to attempt to reference the cultural scope of this paper, and as such, the potential transferability of its findings. Our participants defined themselves as coming from Britain, Australia, Europe, America, Ireland, and Mexico. The paper is therefore embedded within ‘the West’, as the aforementioned countries are considered ‘Western’. These countries are considered to have cultural commonalities which delineate them as separate from other cultures such as the dominance of independent cultural systems and typologies of self-identity (Kirmayer et al., 2018; Muthukrishna et al., 2020).

Typical experiences of grief in the West include an intense longing for the deceased (Uchida et al., 2022), sadness, anxiety, and guilt (Lloyd, 2018), and remaining connected with the deceased through reminders (like feeling the deceased is suddenly present) as well as by internalising aspects of the deceased’s values and identity (Clabburn et al., 2021). Twenty to thirty per cent of the sample in Aoun et al. (2020) (*n* = 908), reported worsening of physical and mental health after bereavement.

As grief is an individual experience (e.g., Sas & Coman, 2016), the present authors would argue, along with Stroebe et al. (2017) or Corr (2019), that categorising grief as ‘normal’ or ‘abnormal’ or understanding grief to progress in a standardised way can be unhelpful, unethical and reductionist. However, when grief becomes worryingly problematic, it can be useful to have something to classify it by. Indeed, Maciejewski et al. (2016:266) explain that for Prolonged Grief Disorder (the grief diagnoses given in the ICD-11 and the DSM-V-TR) and complicated grief (a factor thought to underlie these diagnoses), grief is considered normal, but when symptoms remain ‘too intense for too long’, grief requires clinical attention. In the West, two diagnostic systems are used to diagnose grief ‘disorders’, both using the term ‘Prolonged Grief Disorder’: the DSM-V-TR and the ICD-11. The DSM-V-TR (American Psychiatric Association, 2022) categorises the condition by persistent yearning for the deceased, intense sorrow and emotional pain, preoccupation with the deceased, and preoccupation with the circumstances of the death. The ICD-11’s diagnosis of Prolonged Grief Disorder is similar, encapsulating a longing for or persistent preoccupation with the deceased accompanied by intense emotional pain which causes serious impairment in the individuals’ functioning (e.g., personally, within their family, or at work) (World Health Organization, 2019). As alluded to above, underlying both diagnoses is ‘complicated grief’, where aspects other than grief, for example bereavement-related depression or trauma, interfere with otherwise normal grief processes (Maciejewski et al., 2016). Before the DSM-V-TR (American Psychiatric Association, 2022) updated their diagnosis to Prolonged Grief Disorder, they named it ‘Persistent Complex Bereavement Disorder’. Persistent Complex Bereavement Disorder was estimated to affect 8.2% of the bereaved population (Boelen et al., 2019), and Prolonged Grief Disorder (as defined by the ICD-11) was estimated at 9.8% (Lundorff et al., 2017).

In this paper, the authors consider grief for the death of animals equivalent to grief for the death of humans. Although not always considered in the literature (e.g., Lavorgna & Hutton, 2019), those who experience the death of a pet or companion animal experience loss and grief the same as if the deceased were human (Cleary et al., 2021). Not only do individuals grieve for animals in a ‘normal’ way, but this grief can also become problematic, with those bereaved from animals experiencing prolonged grief, suppressing the grief process, and/or experiencing shame around grief (Kemp et al., 2016).

### The Complexity of Grieving

Grief is often considered ‘taboo’ or ‘hidden’, with conflict arising in navigating grief and bereavement, and grief feeling or being unrecognised. Walter’s (1991:293) discussion about what is taboo or not in ‘modern’ death still holds utility. For example, Graham-Wisener et al. (2022:906) argued that ‘death talk’ was not occurring in adults in community settings and found a lack of acceptance of death and a fear of upsetting oneself or others as barriers to such talk. Walter (1991) argued death was more *hidden* than forbidden and explained that because we have difficulty finding language to talk about death, conversations around it are difficult. Walter (1991) also explained (as have others as cited in Becker, 1973; Ashby, 2017) that as humans, individuals tend towards death denial, inherently moving away from the idea of death the same as anything else which threatens existence or sense of self. Thus, grief and grieving are a complex endeavour because dying, death and bereavement are hidden, lacking in language to describe them, and are topics culturally shied away from.

The complexity of grief is highlighted in the conflict arising within death and bereavement situations. Families can be distressed when other users share the deceased’s private information on social networks (Perluxo & Francisco, 2018). Families can argue over decisions regarding withholding or withdrawing treatment (Breen et al., 2001), and who is considered ‘family’ in these situations (Tilden et al., 1995). Conflict arises in resting places - whether ashes should be scattered in wild spaces (Maddrell, 2010), which form of burial practice should be chosen (Morgan, 2018), and which religious ceremonies should be chosen (Balkan, 2016). Conflict arises in humour, with Wilson et al. (2022, p. 7) reporting that one participant found humour inappropriate when family and friends used this when she “was not ready to laugh”. Conflict and difficult or complicated grief are linked. For example, Mason et al. (2020) found family conflict at end-of-life to be a risk factor for complicated grief. Similarly, Aoun et al. (2020) found that individuals who highlighted lack of support from family and friends (and the community/medical profession) reported the highest deterioration in well-being after bereavement. Thus, it appears that conflict plays a role in grief becoming problematic.

One element arising in grief and bereavement conflict is disenfranchised grief. Explored by Doka (e.g., Doka, 1989; Doka, 1999), disenfranchised grief is a staple concept of today’s grief literature (e.g., Cesur-Soysal & Arı, 2022; Gabay & Tarabeih, 2022), referring to loss which is not openly acknowledged, or mourning processes which are not recognised socially (Attig, 2004). It can arise when a person believes they do not have a right to grieve or to claim social support (Doka, 2008). Disenfranchised grief is also associated with complicated grief, including persistent complex grief disorder and prolonged grief disorder (Adrian et al., 2009). Disenfranchised grief, and the complexities of this, therefore, adds to the picture of why grieving is complex.

### Etiquette

**Figure 1.**
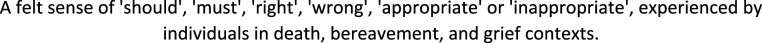
Definition of death, bereavement, and grief ‘etiquette’.

In grief and bereavement contexts, there is a sense of what is and is not acceptable, what should and should not be done, and what, in a given situation, is the ‘right' or ‘wrong' thing to do. This is socially influenced, the social context providing the backdrop for such ‘rights’ and ‘wrongs’. The current article understands this phenomenon under the term ‘death, bereavement, and grief etiquette’. Please see Figure 1 for a definition.

Etiquette can be overt, for example, it would commonly be considered unacceptable to vandalise a burial place. More subtly, etiquette manifests as an embodied ‘felt sense’; an underlying awareness that something is ‘not okay’ or should/should not be done. Importantly, it should be noted that the term ‘etiquette’ describes a phenomenon which has been found from our data; a phenomenon which was identified inductively and needed a name. The phenomenon, rather than the name, came first, with the word ‘etiquette’ chosen as the most accurate fit the authors could find from the English language. Therefore, ‘etiquette’ should be understood by the definition given above. In this context, it is not ‘the formal rules of correct or polite behaviour in society’ (Oxford Learner’s Dictionary, 2023), rather the ‘felt’ or ‘experienced’ rules in death, bereavement, and grief contexts. This distinction is important to avoid confusion.

Although rarely used as a term within the literature, etiquette can be a shaping force in the social norms and experience of grief. Individuals tend to adhere to social etiquette (Barney & Yoshimura, 2020), with literature including multiple examples that demonstrate individuals following how they feel they ‘should’ act. Individuals who had lost a sibling masked their pain from ‘more bereaved’ others and followed an unspoken rule not to mention the siblings’ name (Funk et al., 2018). Non-grieving counterparts displayed responses which were considered over-sympathetic and bereaved individuals put on a “mask” the way they saw others doing (Barney & Yoshimura, 2020, p. 88). Performing for others disenfranchised grief, with grievers forced to attend to the emotions of the over-sympathetic supporter, and requiring the bereaved to ignore their own emotions to create the mask for others. Consequences of contravening ‘etiquette’ can be shock and anger, with Johnson’s (2016) blog post *STOP! Read this before you post another RIP on social media* describing Johnson’s shock when other people posted about their husband’s death on Facebook and their disbelief someone had done so before Johnson had the time to share the news themself. Certainly, we see that etiquette appears to exist in both ‘real life’ and online contexts (coined ‘netiquette’ by Walter et al., 1991), connects with disenfranchised grief, and shapes what we expect of our own and others’ behaviour.

In sum, ‘etiquette’ can be considered to exist and relates to conflict and its consequences in death, bereavement and grief situations. Etiquette is intrinsically bound with disenfranchised grief and those elements known to be risk factors for grief ‘disorders’ and complicated grief. Etiquette as a phenomenon, therefore, warranted further exploration.

This paper reports the first theme from a larger project which asked, ‘where does etiquette arise in death and bereavement contexts and what does this ‘look like?’’ The second theme – *The etiquette of remembrance and values* – will be reported in a separate paper.

## Method

### Philosophical Underpinnings

A social constructive lens was used to identify how participants constructed etiquette within the social context of death, dying and bereavement. A more interpretative lens was then used to consider what etiquette ‘looked like’ and how it was embedded within participants’ experiences. Thus, etiquette was understood as both socially and linguistically situated (Pilgrim, 2014), being embedded in both the social world and reported through the merits and limitations of language.

### Recruitment and Participants

Purposive and opportunity sampling was used (Campbell et al., 2020), with adverts placed on social media or emailed by gatekeeper organisations (Cruse Bereavement Care, Radio Royal Bradford, Sheffield Death Group, Bath University’s Centre for Death and Society). Although seven of the participants were grief professionals, the adverts clearly stated the researchers wanted to interview individuals about their personal experiences of death, dying, and bereavement etiquette. Excepting one individual, all participants spoke from a personal perspective first and a professional perspective second (if at all). Indeed, many of the participants who had become grief professionals had done so after (later than) the experiences they shared during their interview.

Once participants indicated interest, they were sent an information sheet and consent form followed by a demographic questionnaire. The demographic questionnaire collected both contextual information about the participant (e.g., age, gender) and information concerning the context of their bereavement (e.g., age at the time of death, relationship to the deceased). As well as being useful for situating the findings of the study (Brinkmann & Kvale, 2018), this information allowed interview questions to be personalised, resulting in richer data and rapport being developed earlier (Willig, 2008).

Prior to participation, participants received information pertaining to the aims of the study through the information sheet. They were given the definition of etiquette included in this paper’s introduction (see Figure 1), and told the researchers were interested in ‘the rules’ around death and bereavement - what ‘rules’ individuals hold in these situations and what is it like to experience them. For example, how do individuals feel about memorialising the deceased on social media? What do they think we should do with people’s belongings after they have died? How often should we visit gravesites? We explained to them that we wanted to get a clearer picture about these topics, ones we don’t often talk about.

Participants were bereaved individuals (*n* = 4), grief professionals (e.g., funeral director, *n* = 1), or both (*n* = 6), allowing us to understand death and bereavement etiquette from multiple perspectives (Robinson, 2014). Although we understood that discussing bereavement may be upsetting, we asked individuals who thought they might become *distressed* not to take part. Consistent with the British Psychological Society’s Code of Human Research Ethics (BPS, 2021), the information sheet explained how/when participants could withdraw (any time during and up to two weeks post-interview, no participants withdrew), data handling, and whom to contact for further information/complaints. Eleven interviews conducted and recorded on MS Teams by experienced qualitative researchers JD (DCounsPsych, Senior Lecturer in Psychology, female) and JK (PhD, Senior Lecturer in Psychology, male) between April to June 2021 ranged between 52 and 86 minutes and provided sufficient information to answer the research question (Braun & Clarke, 2021a). Please see Table 1 for a tabulated representation of our participants.

**Table 1.** Collating Participants’ Demographic Information.

Demographic	Detail	Number of participants
Age	25–34	4
	35–44	3
	45–54	1
	55–64	2
	65–74	1
Gender	Female	7
	Male	4
Ethnic background	Caucasian Australian	2
	Mexican	1
	White British	5
	White European	1
	White American/Irish/British	1
	White American	1
Religious or spiritual beliefs	Spiritual	5
	Agnostic	2
	Atheist	3
	Agnostic and atheist	1
Position at time of interview	(Speaking as) personally bereaved	4
	(Speaking as) a professional	1
	(Speaking as) both personally bereaved and a professional	6
Type of bereavement^a^	Bereaved by person	5
	Bereaved by both person and pet.	5

^a^This excludes the one person speaking just from professional experience.

### Data Collection

Before the interview, a topic guide was developed by the researchers including the subjects of social media, treatment of the dead body, resting sites, supporting the bereaved, communication with the dying, and objects. Specific questions were then developed to gather data on these topics based on participants’ responses to the demographic questionnaire. Participants were informed that the interview would focus on ‘etiquette’ and, as mentioned earlier, were given the same definition outlined in the introduction (see Figure 1).

The semi-structured question schedule included prompts like ‘can you tell me more about that’ and allowed interviewers to focus on listening rather than talking (Galletta, 2013). Notes were made throughout but acted simply as an aid memoir and were destroyed afterwards. Interviews began with a warm-up question to build rapport (Willig, 2008), important for the sensitive nature of the topic (Newcomer et al., 2015), and employed a traffic light system to safeguard emotion where participants or the interviewer would communicate how they were feeling (green = emotionally fine and would like to continue, amber = feeling emotional but would like to continue, red = feeling emotional and would like to change the topic or end the interview).

### Analysis

JD, JK, and MS (BSc in Psychology with Counselling and Psychotherapy, Research Assistant, male) coded and developed themes and subthemes following Braun and Clarke’s (2006) reflexive thematic analysis. After JD had written the narrative, JK and MS reviewed this for triangulation (Heale & Forbes, 2013). A relationship between the researchers was developed in which individuals were reflexive and open, ‘arguing the case’ or not for codes or themes, and suggesting data be discarded if it was felt too influenced by the interviewer’s personal perspective (O’Reilly & Parker, 2012). This reflexive approach enhanced the credibility and authenticity of the interpretations (Braun & Clarke, 2021b).

An inductive ‘bottom up’ approach allowed new and unexpected insights (Braun & Clarke, 2006) and for complexities to be explored (Braun & Clarke, 2021c; Clarke et al., 2015). Themes were identified as groupings conveying key answers to the research question, consistent throughout the dataset (Braun & Clarke, 2019). Semantic themes allowed us to best generate a rich description of the data set however meant some complexity was lost (Braun & Clarke, 2006). To counteract this, we have explored the data through different methods (e.g., interpretative phenomenological analysis, Smith et al., 2022) to be submitted for publication later. Consistent with Braun and Clarke (2021a), saturation at the level of the area (where etiquette arises and what it looks like) was not considered possible or achieved, with saturation not being used to determine sample size or when data collection was stopped. However, the researchers were guided by information power to determine when they had enough data to respond to their research question (Braun & Clarke, 2021a). Transcripts were not returned to participants for correction although each was reviewed by multiple members of the team and summaries were sent to participants to inform them of the study’s findings (BPS, 2021).

### Ethical Considerations

Manchester Metropolitan University’s ethics board approved the study (ID: 23827) and the researchers followed the university’s ethical framework (MMU, 2021). Confidentiality has been maintained as much as possible via pseudonyms, changing identifying identification, and by ‘chunking’ demographic information so not to reveal identities to those who know participants well (BPS, 2021). Data was kept securely on an encrypted server (Kaiser, 2009). Unless participants requested to be kept abreast of findings and publications, all personal data (names/email addresses) has been destroyed in line with GDPR frameworks. Though we endeavoured to minimise distress, support organisations were included on the information sheet for immediate access following interview. Finally, Willig (2013) notes that interpretation is always a transformation process, affording the researchers power over how readers respond to and understand others’ truths. In this way, the findings should be understood as *an* interpretation rather than *the* interpretation of the data.

## Findings and Discussion

**Figure 2.**
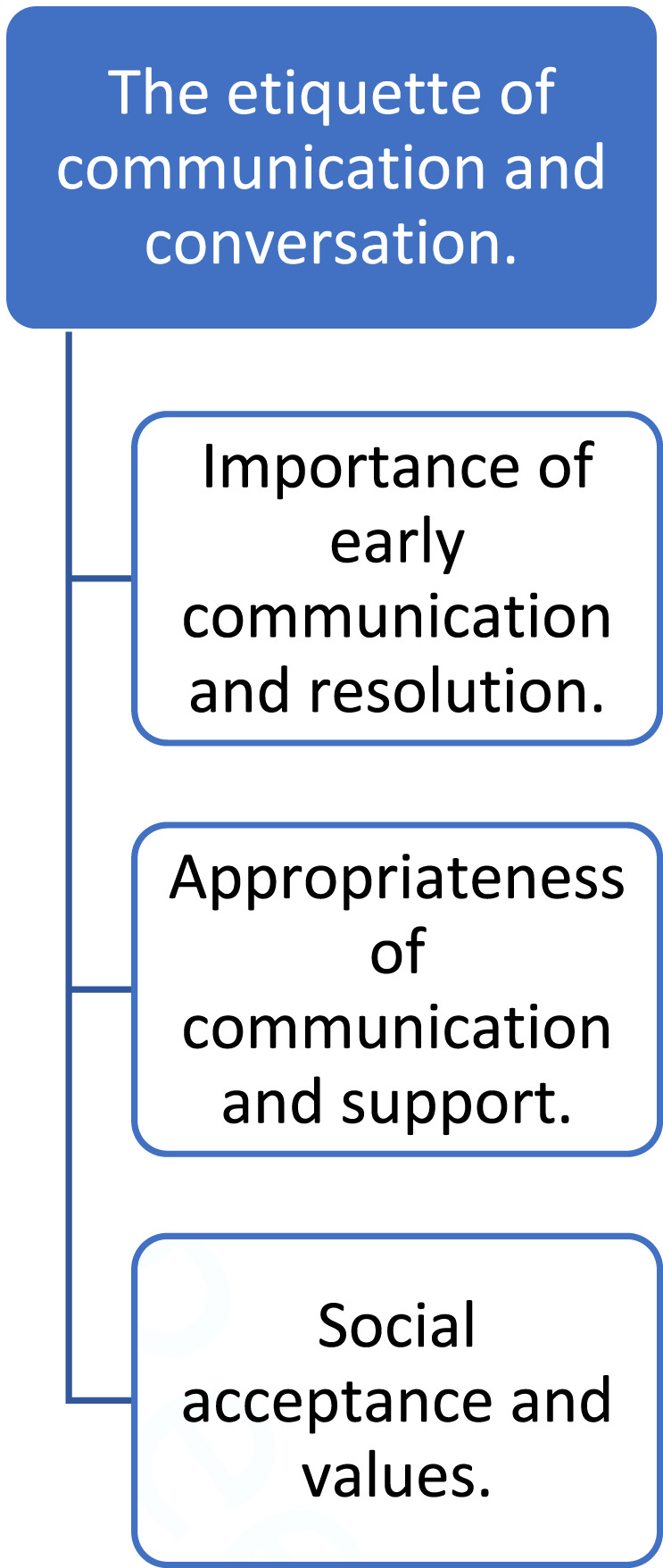
Coding tree of theme the etiquette of communication and conversation.

Although ‘etiquette’ is a phenomenon which may exist within many cultures, it should be noted that the following themes, their discussion, and the resulting guiding principles are drawn from a Western cultural context. Thus, they may only be relevant to the West and should be read as such. Analysis created a rich set of themes representative of the participant group. Etiquette was present within communication and conversation, with early communication considered important to reduce and resolve the potential for conflict and distress. This was expected to be ‘appropriate’, meaning present, sensitive, and thoughtful. What was considered sensitive and thoughtful was influenced by social values and what, on the other hand, society deemed unacceptable. Please see Figure 2 for a coding tree of the theme *The etiquette of communication and conversation.*

### The Etiquette of Communication and Conversation

“For a really long time I’ve been thinking, ‘why didn’t they have the conversations? Why didn’t they feel they could say whatever they wanted or needed to say before it was too late?’” Nom De Plume, lines 151–152.

### Subtheme 1. Importance of Early Communication and Resolution

Communication and resolution were spoken about across the lifespan; in the context of the young, when death was on the horizon, and in the days before dying. Preparation for death (and therefore conversations around e.g., wills, property, and funeral arrangements) was considered extremely important, borne from participants’ experiences of when death had not been prepared for, either because it was unexpected (e.g., Clara) or because those conversations had simply not occurred.

Participants described an awkwardness around conversations, with death being a topic reported to make others uncomfortable in general, and when it came to navigating practical issues such as property. This awkwardness meant conversations had not happened, or had been delayed, and because resolutions had not been known or found, participants were caused stress and emotional difficulty.

For example, Laurel described how, with no prior communication, organising her uncle’s funeral felt stressful, almost burdensome:

“Thankfully [my uncle’s wife] came back to her senses in time for the funeral. [] At that point, the weight that lifted off my shoulders was immense. I didn’t even realise the weight I was carrying until my Mum rang me and said, ‘I’ve gone through the funeral plan with Michelle, [your uncle] had already written what he wanted’.” Laurel (lines 289–293).

Whereas the emotional difficulty was eased for Laurel when prior communication and its resolution was communicated to her, Cerys and Judith reported different experiences. Cerys felt angry towards her estranged mother for not reaching out to reconcile before she died. There was a sense that Cerys’ mother robbed her of this opportunity by dying:

“When she was alive, there was always hope that she would… [] there would be some apology. I feel like I was always a good daughter. [] When she died, I was really angry with her, because she took that [chance at reconciliation] away from me.” Cerys (lines 99–105).

In Cerys’ account, there was a sense of ‘should’. Cery’s mother ‘should’ have reached out. This lack of communication and resolution led to emotional difficulty.

The findings align with the cognitive-behavioural (CBT) theory of distress, whereby thoughts and beliefs are understood to cause and maintain distress rather than situations themselves (Trower et al., 2007). As exemplified above, the etiquette underpinning participants’ distress when, for example, choosing flowers or music for a funeral, was that there was a ‘right thing’ to do and concern that individuals had not done what they ‘should’. Setting aside criticisms of CBT theory (e.g., Moloney & Kelly, 2004; Naeem et al., 2019), data gave insight into etiquette-based thoughts which feed distress and could be worked with in this popular (e.g., Cuijpers, 2017) therapeutic method.

Specifically, data highlighted how the unhelpful thinking style *‘shoulding and musting’* might manifest in death, bereavement, and grief situations. Unhelpful thinking styles (or ‘dysfunctional assumptions’ - Beck, 1976) are common and automatic ways of thinking which create and maintain distress (e.g., Leahy & Holland, 2000). ‘Shoulding and musting’ involves individuals telling themselves they ‘should’ or ‘must’ do something resulting in unrealistic expectations for the self and others (Centre for Clinical Interventions, 2019). These thoughts of ‘should’ and ‘must’ were rife within our data (see Table 2 for examples).

**Table 2.** ‘Should’ and ‘Must’ Thoughts Fuelled by Etiquette.

I should put my grief to the side if others are more upset than me	I should choose music and flowers for a funeral which the deceased would have liked, and which represents them
The deceased should have a physical resting space with their name on it	People should reach out to reconcile with family if they know they are dying
The eulogy should accurately represent the deceased	People should discuss plans for the euthanasia of pets with the people the pets belong to
I should know where the remains of my pet are	Even though it is uncomfortable, people should talk about what they want for their funerals with those who will be organising them

CBT has its own methods to target unhelpful thinking styles, namely challenging these (e.g., Greenberger et al., 2015), whereby alternative thoughts and evidence are considered. Although identifying ‘should and must’ thoughts and working with these is important to avoid or reduce distress and disenfranchisement, it is beyond this paper’s scope to discuss CBT application further. We trust that trained practitioners can see the utility of the theory and could follow the CBT model (or another model) to provide intervention.

All participants who experienced emotional fall-out from lack of communication (*n* = 6) took an ‘ambassador’ role later, pushing forward conversations about death and grief either professionally or in their personal lives. For example:

“I will bleat on [] to my [immediate family] and say, ‘if it matters to you, what we play at your funeral, [] write it down’. [] I’ll say this all the time. ‘If you have specific flowers that you want [] make them known.’” Laurel (lines 296–300).

Participants aimed to push through awkwardness to ensure early communication and early resolution, thus avoiding emotional difficulty for their future selves and others. Although conversations were often uncomfortable, they felt essential, the pro of the conversation (resolution) outweighing potential cons (e.g., awkwardness, upsetting others).

**Figure 3.**
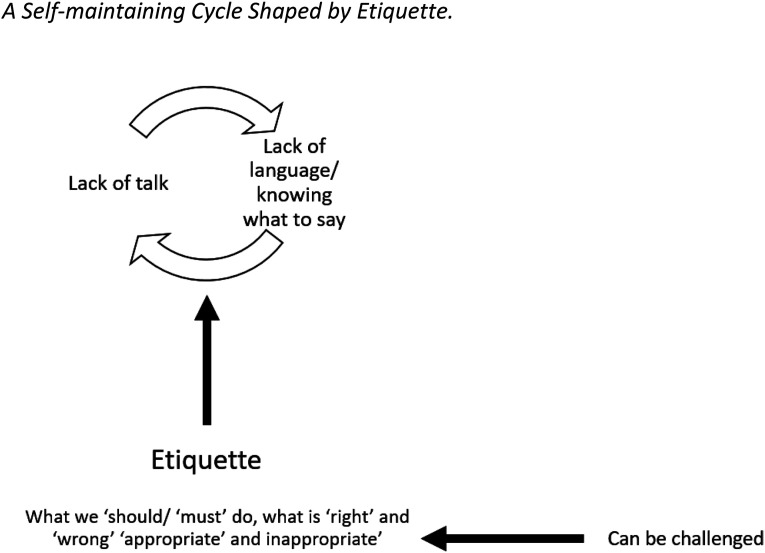
A self-maintaining cycle shaped by etiquette.

The study identified dialogues which, when absent, contributed to disenfranchised grief. Processes such as having a gravestone (Jayne), making a living will (Clara), and expressing grief over pets (Jerry, Judith, and Laurel) were identified as topics seldom discussed. This data adds to Walter’s (1991) claim that trouble finding language to talk about death renders conversations difficult (and less likely to occur). There appeared a self-maintaining (or ‘vicious’ – Greenberger et al., 2015) cycle, whereby not talking led to a lack of language or knowing what to say, which led to not talking (see Figure 3). Etiquette shaped what participants talked about and whether topics were brought up (again, see Figure 3). This leads to two considerations. First, as described by Burr (2015), having terms to describe something allows us to talk about it, therefore the term ‘etiquette’ is helpful because it allows the ‘shoulds’ ‘musts’ ‘rights’ ‘wrongs’ ‘appropriate’ and ‘inappropriate’ in death and bereavement contexts to be discussed, considered, and challenged (see Figure 3). Second, in considering and challenging etiquettes, a wider variety of conversations can be had, providing a method of intervening in this self-maintaining cycle. Thus ‘etiquette’ - what it is, where it arises, and what it consists of – may be a valuable part of grief education and ultimately enfranchising grief.

### Subtheme 2. Appropriateness of Communication and Support

Participants (Laurel, Jerry) expressed that the most appropriate way to learn about death was in person. Inappropriate methods felt impersonal and lacked consideration of the bereaved’s emotions. Via text message, with little context about the illness or death, felt inappropriate, as were impersonal posts on Facebook. Participants were left shocked, stunned, and upset when they learned about their loved ones’ deaths in these ways.

Where Facebook had to be used to inform of a death, direct message – something private and personal – was felt more acceptable. However, accessing the deceased’s Facebook account brought etiquette considerations of its own, feeling like an invasion of the deceased’s privacy:

“I managed to log into [my brother’s] laptop and figure out his passwords. [] Then I contacted [his friends] through my account. Because we weren’t friends, there’s another inbox on Facebook so [one guy] didn’t get the messages. I really needed to let him know [] so I [] message[d] him from my brother’s account. That felt a bit weird. It felt like an intrusion in my brother’s privacy.” Faye (lines 283–296).

Once the death had been communicated, etiquette permeated individuals’ responses. Those who had been informed were expected to contact the bereaved, and in instances when the death had not been acknowledged, the bereaved felt uncomfortable and ‘off-footed’. The ‘right’ thing to do was to prioritise the bereaved’s needs, imagining what they might be experiencing and, consequently, not pushing for information they might not feel ready to give. Certainly, it was unacceptable to treat the death as a source of entertainment (which one participant had experienced due to a high-profile death). Where individuals contravened these rules, the bereaved felt under pressure, out of control, and like who they were as people – and who their loved ones were – was not as important as the story’s drama. For example:

“[I had] really bad interactions where I would say, ‘my friend was killed’ [] and [] people would say, ‘what happened’ and it was the entertainment. They didn’t care how I felt or who she was or what she meant to me. They wanted to know how she was killed and how [the police] found the guy who did it. That’s terrible.’’ Clara (1115–1133).

When supporting the bereaved in general, getting in touch and offering support was considered important, with phone calls and messages an appropriate form of communication unless the bereaved were very close family/friends who would be left lonely by their bereavement. Here, being physically present was considered best. For Faye, when her friend did not get in touch to offer support, the friendship broke down, leading her to re-evaluate its merit. It appeared that the ‘right’ level of communication was dictated by the closeness of the relationship pre-bereavement, with close friends/family expected to continue this closeness in the support offered.

Some participants had ‘hard and fast’ rules regarding communication post-death. Clara always wanted others to acknowledge her loss regardless of whether it was awkward or made her upset, and Faye did not want people to message her about the death whilst at work. Cerys emphasised the importance of the bereaved clearly communicating what they wanted and needed. In this way, confusion about how others should respond would be reduced, and supporters could avoid contravening the expectations or rules the bereaved held for them:

“If we were [] more honest about what we need and we asked for what we needed [] then I think other people would be able to do the ‘woulds’ and the ‘shoulds’, the ‘ifs’ and the ‘buts’.” Cerys (lines 354–356).

Cerys’ account gave insight into helpful conduct for the bereaved. In particular, she highlighted the importance of educating the bereaved about how they could help others help them. Although literature (e.g., Hill & O’Brien, 2021; Meisenhelder & Gibson, 2015) provides guidelines for supporting the bereaved, less emphasis is placed on (a) what the bereaved can do to elicit the support they need and (b) how they can best navigate their grief with others. As Cerys argued, we need to help others help us. Future research could focus on gathering the perspectives of those supporting the bereaved, or reviewing the available literature, to create guidelines for the most helpful conduct from the bereaved (much like a reversed version of Meisenhelder and Gibson’s (2015) work providing guidelines on how to care for bereaved parents).

As well as ‘dos’, participants described ‘donts’ when supporting the bereaved. It was not okay to tell people what to do (e.g., to sue the hospital) or to “put [] their grief” (Faye, line 103) on others. The latter might manifest in individuals ‘holding grief in’ so the bereaved are not positioned to ‘hold’ others’ distress or comfort them when dealing with their own difficulty.

Data provided guidelines on what to do if you learn of the death of someone you know. Understanding what to do was not always obvious (e.g., Judith), and not receiving expected assistance resulted in relationships failing (Faye). Such experiences have been described in the literature, with people being frequently unclear about what to say or do in mourning circumstances (Graham-Wisener et al., 2022) and relationships breaking down when expected assistance is not provided (Popoola et al., 2021). Each person and each situation is different therefore, we would avoid suggesting ‘hard and fast’ rules as well as exercising a certain level of social empathy. Following the precedent set by other literature (e.g., Hill & O’Brien, 2021; Meisenhelder & Gibson, 2015) who provide guidelines for supporting the bereaved, this paper offers the below flexible principles guided by its data and findings (please see Table 3).

**Table 3.** Potential Guiding Principles for Supporting the Bereaved.

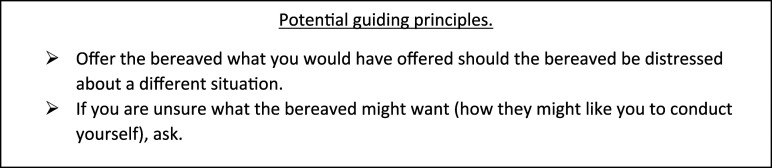

When used with sensitivity to the situation, adhering to these principles may enfranchise grief, as supported by Aoun et al.’s (2020) and Mason et al.’s (2020) findings on the importance of social support for ‘healthy’ grief.

### Subtheme 3. Social Acceptance and Values

Participants' reports of death conversation, pet loss, and how they felt able to grieve were influenced by societal standards about what was (or was not) socially acceptable. Cerys emphasised that people lack “grief literacy” (line 365) - the ability to articulate oneself, ask for what one needs, and understand what is normal. Lack of grief literacy appeared partly caused by lack of communication about mourning processes, with participants unable to learn from those around them about what was ‘normal' and hence make decisions based on this. Discussing gravestones, advanced directives, living wills, and funeral processes was considered helpful to learn about these and what might be common practice regarding them. As Jayne explained:

“You don’t talk to people, do you? You don’t say, ‘well, how long til you got a gravestone?’ Or, ‘do you have one?’ ‘Does everyone in the family have one?’. You just don’t know [if all people in a family have a gravestone] because some people might not.” (Jayne, 166–168).

The etiquette – the sense of talking about gravestones and living wills as ‘not the done thing’ – disenfranchised participants in the death and bereavement space. In not knowing what was ‘normal’, Jayne was left feeling impotent to pursue conversations with her father about getting her mum a gravestone.

We can see how lack of communication and resolution, and lack of appropriate communication and support, could be risk factors for disenfranchised/complicated grief. Above, Jayne speaks about the lack of communication about gravestones and, thus, a lack of resolution. Data highlighted other situations like not knowing whether the ‘right' music or flowers were chosen for the funeral (Laurel), not resolving relationship difficulties before death (Cerys, Nom De Plume), and not knowing where the deceased’s remains were (Judith) to create difficulty and distress. These findings align with Mason et al. (2020), who report conflict around death as linked to difficult or complicated grief. Similarly, participants described learning of death in impersonal ways or not having their loved one’s death recognised as at best uncomfortable (Laurel), and at worst, traumatic (Jerry). The literature would benefit from further research to explore these links more nuancedly in samples experiencing complicated grief or who meet the criteria for grief diagnoses.

Grieving for a pet was also reported to be disenfranchised. Interestingly, grieving rules were not just overt – for example, Judith’s father’s stance that “a man in his 60s would never grieve a dog” (line 248–249) – but sensed by society also. Although Jerry had not encountered direct prejudice because of his dog’s death, he predicted others would “not have understood and [] judged [my grief] in a negative manner” (lines 358–359), discouraging him from talking about his loss. The anticipated judgement and the sense that others would consider his grief unacceptable in some way led to this being disenfranchised.

Rather than individuals being free to navigate death and grief in their own way, expectations (such as established customs like religious services or thoughts about what the ‘right’ thing to do was) were either placed on individuals or sensed by them. When individuals did not meet these expectations, the ‘acts of omission’ were considered to mean something, as in Judith’s report of her cousin:

“When [my uncle] died [] my cousin wouldn't go to the hospital. She wouldn't have any say in turning off life support or anything like that. [] A lot of people held onto resentment about that.” Judith (lines 66–69).

Judith explained her family were angry her cousin had not gone to see her uncle when he was dying. Not going seemed to indicate something, some measure of disrespect. There could have been many reasons why Judith’s cousin did not go. From other’s accounts (e.g., Jerry), it seems likely this was not felt the best course of action by Judith’s cousin at the time. We can see that such expectations – from the family, borne from societal values about seeing the dying – set parameters around the individuality afforded to individuals navigating death situations and in their grieving.

## Conclusion and Future Directions

The findings align with the CBT understanding of distress with data highlighting how ‘shoulding and musting’ might manifest in death and bereavement contexts. Data also gave insight into conversations that, when missing, might lead to disenfranchised grief. Etiquette underpinned the likelihood that conversations might occur and how they might progress, providing rationale for etiquette to be considered in grief literacy education. Usefully, the data offered suggestions for supporting the bereaved and provided helpful actions the bereaved could take to gain the input they felt was required. Findings further indicated that a lack of communication and resolution and a lack of appropriate communication and support – underpinned by etiquette – could be risk factors for disenfranchised/complicated grief. We suggest future research builds on our work to explore exact conversations missing in samples of those meeting criteria for grief diagnoses. Although we acknowledge that a better word could be found, the term ‘etiquette’ provided language to discuss the nuances of bereaved people’s experiences and was a concept which appeared to resonate with the bereaved community.

### Strengths and Limitations of the Current Study

Employing the term ‘etiquette’ expressed an idea experienced by participants, allowing them to discuss it. Thus, the research addressed Walter’s (1991) assertion that we lack language to discuss death. Etiquette appeared to resonate with the bereaved community and recruitment was swift. Thus, etiquette appears a phenomenon which resonates with people experiencing loss and assists them in describing and discussing their experiences.

The research team acknowledge that a better term to ‘etiquette' could be used. Etiquette might be (mis)interpreted to mean a set of rules individuals should follow rather than a term to describe an observed phenomenon. Similarly, etiquette is comprised of both explicit norms and implicit felt sense. Whereas more overt rules can be argued and disputed since people are aware of them, a felt sense is just that – a sense of something without clear thought or understanding. Perhaps further work could explore the use and functions of language by individuals who are bereaved to better define etiquette and the sub-types within this context.

Furthermore, the authors acknowledge that the research and papers cited in context are predominantly situated and shaped by ‘Western’ cultural values. As such, the transferability of the findings may be limited to understanding death, dying, and bereavement from a ‘Western’ perspective.

In conclusion, this is the first of two papers that describe data addressing the question, ‘Where does ‘etiquette' arise in death and bereavement contexts, and what does this ‘look like?’’ The theme Etiquette of Communication and Conversation was developed through reflexive thematic analysis. Early communication was critical in reducing and addressing the possibility of conflict and distress. Communication and support were expected to be ‘appropriate', meaning present, sensitive, and considerate. What was judged sensitive and considerate was shaped by societal standards and by what society deemed objectionable.
